# Mammographic Breast Density and Breast Cancer Molecular Subtypes: The Kenyan-African Aspect

**DOI:** 10.1155/2018/6026315

**Published:** 2018-01-22

**Authors:** Asim Jamal Shaikh, Maeve Mullooly, Shahin Sayed, Rose Ndumia, Innocent Abayo, James Orwa, Ronald Wasike, Zahir Moloo, Gretchen L. Gierach

**Affiliations:** ^1^Section of Medical Oncology, Department of Medicine, The Aga Khan University Hospital, Nairobi, Kenya; ^2^Division of Cancer Epidemiology and Genetics, National Cancer Institute, National Institutes of Health, Bethesda, MD, USA; ^3^Cancer Prevention Fellowship Program, Division of Cancer Prevention, National Cancer Institute, Bethesda, MD, USA; ^4^Department of Pathology, The Aga Khan University Hospital, Nairobi, Kenya; ^5^Department of Radiology, The Aga Khan University Hospital, Nairobi, Kenya; ^6^Department of Medical Education, The Aga Khan University Hospital, Nairobi, Kenya; ^7^Department of Statistics and Epidemiology, The Aga Khan University Hospital, Nairobi, Kenya; ^8^Section of Breast Surgery, Department of Surgery, The Aga Khan University Hospital, Nairobi, Kenya

## Abstract

**Introduction:**

Data examining mammographic breast density (MBD) among patients in Sub-Saharan Africa are sparse. We evaluated how MBD relates to breast cancer characteristics in Kenyan women undergoing diagnostic mammography.

**Methods:**

This cross-sectional study included women with pathologically confirmed breast cancers (*n* = 123). Pretreatment mammograms of the unaffected breast were assessed to estimate absolute dense area (cm^2^), nondense area (cm^2^), and percent density (PD). Relationships between density measurements and clinical characteristics were evaluated using analysis of covariance.

**Results:**

Median PD and dense area were 24.9% and 85.3 cm^2^. Higher PD and dense area were observed in younger women (*P* < 0.01). Higher dense and nondense areas were observed in obese women (*P*-trend < 0.01). Estrogen receptor (ER) positive patients (73%) had higher PD and dense area than ER-negative patients (*P* ≤ 0.02). Triple negative breast cancer (TNBC) patients (17%) had lower PD and dense area (*P* ≤ 0.01) compared with non-TNBCs. No associations were observed between MBD and tumor size and grade.

**Conclusions:**

Our findings show discordant relationships between MBD and molecular tumor subtypes to those previously observed in Western populations. The relatively low breast density observed at diagnosis may have important implications for cancer prevention initiatives in Kenya. Subsequent larger studies are needed to confirm these findings.

## 1. Introduction

Mammographic breast density (MBD) is defined as the relative amounts of radio dense stromal and epithelial tissue compared with radiolucent adipose tissue. MBD is recognized as an established independent breast cancer risk factor [1]. It is a heritable trait [2, 3] and is thought to reflect cumulative exposure to established breast cancer risk factors, including parity, age at first birth, age at menopause, and endogenous and exogenous hormonal influences [4–7]. Studies of primarily Western populations have suggested that elevated MBD is a general marker of breast cancer risk, irrespective of molecular tumor subtype [8]. In addition, elevated MBD has been associated with adverse breast tumor clinical characteristics including larger tumor size, nodal involvement, and advanced stage at diagnosis [1, 6, 9]. Relationships between MBD and adverse prognostic features may reflect decreased mammographic sensitivity in women with dense breasts, resulting in delayed diagnosis in screened populations [6]. Furthermore, among women diagnosed with breast cancer, MBD may play a role in breast cancer progression [10]. However, much of the MBD evidence published to date is largely based on studies carried out among Western populations and it is currently unclear whether these findings are similar among African women.

Little is known about MBD within Sub-Saharan Africa (SSA), particularly among indigenous women. One study of 190 Ugandan women without breast cancer, referred for screening, with an average age of 46 years (standard deviation (SD) 13 years) at the time of mammography, indicated possible differences in patterns of MBD from those observed in the United States (US) or Europe, with a higher proportion of study participants having low MBD [11], as defined visually in categories using the American College of Radiology's Breast Imaging Reporting and Data System (BI-RADS) [12]. The proportion of Ugandan women with low MBD (BI-RADS category 1) was 41% [11], which is greater than the proportion of US women in this lowest BI-RADS density category, which ranges from 0.6 to 9.4%, depending on race, age, and body mass index (BMI) [13, 14]; BMI was not assessed in the Ugandan report [11]. Whether differential patterns of MBD exist and how these MBD patterns relate to breast tumor characteristics in other regions of SSA is unknown.

In particular, the role of MBD in relation to breast cancer clinical characteristics within Kenya is currently unclear. To investigate this, we performed a cross-sectional analysis, examining MBD among indigenous Kenyan women with breast cancer utilizing quantitative software for MBD assessment. We examined relationships between MBD with features of breast cancer aggressiveness, including breast molecular tumor subtype and clinicopathological breast cancer characteristics.

## 2. Methods

### 2.1. Study Population

This study was a cross-sectional study of indigenous Kenyan women with pathologically confirmed breast cancer, diagnosed between February 2014 and May 2015 at the Aga Khan University Hospital, Nairobi. Diagnoses were confirmed by a breast pathologist, according to the National Comprehensive Cancer Network® (NCCN®) guidelines, which are followed as institutional policy for classification and treatment related decisions. Overall, 123 women out of 148 who were diagnosed with breast cancer during this time-period had pretreatment, diagnostic mammograms available for assessment. Participant information was abstracted from medical records and a computerized database included patient age, menopausal status, and BMI. Upon diagnosis, all breast cancer biopsies underwent a central pathology review with double reporting of clinical and tumor characteristics, including tumor size, tumor type, grade, and lymph node metastases. Breast cancer molecular subtype was defined according to estrogen receptor (ER)/progesterone receptor (PR) and human epidermal growth factor receptor 2 (HER2) status, which was evaluated using immunohistochemistry on the Dako Automated platform followed by pathology review (ZM/SS). Interpretation of ER/PR was done according to the Allred scoring system [15]. HER2 scoring was done according to the American Society of Clinical Oncology (ASCO)/College of American Pathologists (CAP) guidelines. Samples that scored as HER2 2+ (equivocal) were subjected to fluorescence in situ hybridization (FISH) testing to assess gene amplification, at the Aga Khan University Hospital reference laboratory in Karachi Pakistan. A FISH test result was considered positive if ratio of the HER2/neu/CEP17 signals was >2.2 [16].

All patients were consented for utilization of their information in this study. Approvals to conduct this analysis were obtained from University Research Ethics Committee prior to the study commencement.

### 2.2. Assessment of Mammographic Breast Density

MBD was assessed by a single radiologist (RN) from diagnostic digital mammograms (mediolateral view) of the unaffected breast, taken prior to any treatment was instituted, and using the semiautomated Cumulus 4 Software [17], a well-studied quantitative method of MBD assessment [6]. Cumulus is a computer-assisted thresholding program in which the user defines a threshold for the skin edge and dense area. Three independent measures of MBD were determined including percentage density (PD), absolute dense area (cm^2^), and total breast area (cm^2^). PD was calculated by dividing the dense breast area by the total breast area and multiplying by 100. Nondense area (cm^2^) was calculated by subtracting absolute dense area from the total breast area. The radiologist was masked to demographic and pathologic data. Quality assurance and reliability assessment of MBD measurements were performed internally for the first 25 cases using visual assessment by a second radiologist at the Aga Khan University Hospital, Nairobi. This independent assessment showed 97% concordance for PD > 50%.

### 2.3. Statistical Analysis

To examine the relationships between quantitative MBD measurements and patient and clinical characteristics, we used analysis of covariance (ANCOVA) models. Characteristics investigated included age (<50 years, ≥50 years), menopausal status, BMI (kg/m^2^ classified according to the World Health Organization as lean, overweight and obese) [18], histology (invasive ductal vs. other), tumor size (<3 cm, ≥3 cm), tumor grade (grade 1, grade 2, grade 3, and unknown), nodal status (negative, positive or unknown), ER status (negative, positive, unknown), PR status (negative, positive, and unknown), HER2 (negative, positive, and equivocal/ductal carcinoma in situ (DCIS)), and triple negative breast cancer (TNBC) status [no, yes (i.e., negative for ER, PR, and HER2), and unknown]. MBD measurements were square root transformed to better approximate normal distributions. For ease of interpretation, the least square means and standard errors from ANCOVA models were back transformed, and corresponding 95% confidence intervals were calculated and are presented in the tables. All models are presented as unadjusted and adjusted for age and BMI. The results of adjusted models are discussed below unless otherwise mentioned. In sensitivity analyses, investigations of relationships between MBD measurements and molecular tumor subtypes were also stratified by menopausal status. *P* values of <0.05 were considered statistically significant and tests of statistical significance were two-tailed. Reported *P* values were not corrected for multiple testing. All analyses were performed using SAS software v9.3 (SAS Institute Inc., Cary, NC).

## 3. Results

### 3.1. Overview of Study Population

Patient demographic and breast tumor clinical characteristics for the overall study population are presented in Table 1. Participant characteristics stratified by ER status are also shown in Table 1. The median (range) age at diagnosis was 54 (27–83) years, with most women being postmenopausal (66%) and overweight/obese (79%) at diagnosis. Most breast tumors were invasive ductal (85%), ER-positive (73%), PR-positive (67%), and HER2-negative (76%). The median (range) tumor size was 3 cm (0.5–10 cm). A higher proportion of participants had grade 2 tumors (46%) and was positive for lymph node metastasis (49%). Compared with ER-positive breast cancers, ER-negative tumors tended to be larger, higher grade, and PR-negative (Table 1). No significant differences were observed between ER-positive and ER-negative breast tumors for patient age, menopausal status, BMI, nodal status, and HER2 status (Table 1).

### 3.2. Relationships between MBD Measurements and Patient and Clinical Breast Tumor Characteristics

**Figure 1:**
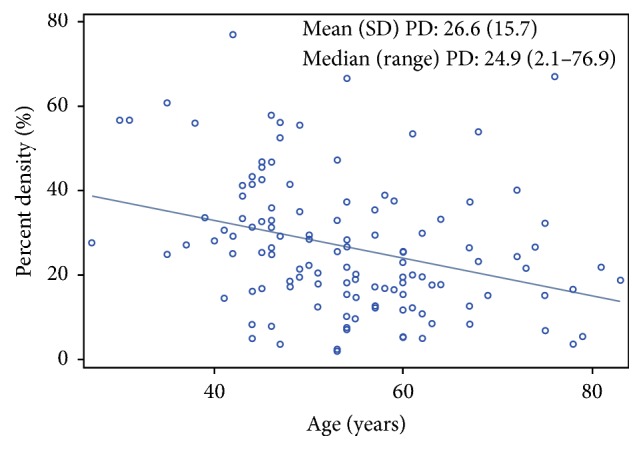
Distribution of mammographic percent breast density (PD) measurements by age.

The distributions of MBD measurements by age are shown in Figure 1 and Supplementary Figure 1. The median (range) PD was 24.9% (2.1–76.9%) and median (range) dense area was 85.3 cm^2^ (1.5–355.6 cm^2^). Relationships between MBD measurements and breast tumor clinical characteristics are shown in Table 2. On average, significantly higher MBD was observed in younger (<50 years) versus older (≥50 years) women (adjusted mean PD: 31.8 versus 19.8%, *P* < 0.01; adjusted mean dense area: 110.1 versus 72.1 cm^2^, *P* < 0.01). Significantly lower nondense area was also observed in younger than older women (232.5 versus 306.9 cm^2^; *P* < 0.02). In unadjusted models, higher PD and lower nondense area were observed in pre- versus postmenopausal women (*P* < 0.01); however, these findings were attenuated after adjusting for age and BMI. Significantly higher dense area and nondense area, reflecting higher total breast area, were observed in women who were obese versus overweight/lean (*P* values for trend < 0.01 for each); however, relationships between PD and BMI were not statistically significant. No relationships were observed between the MBD measurements examined (PD, dense area and nondense area) and breast tumor, size, grade, and nodal status (Table 2).

### 3.3. Relationships between MBD Measurements and Breast Molecular Tumor Subtype

Unadjusted and adjusted relationships between MBD measurements and breast molecular tumor subtype are shown in Table 3. In addition, because prior studies have shown that the prevalence of dense breasts as well as breast cancer molecular subtypes vary by menopausal status, we carried out sensitivity analyses investigating relationships between MBD measurements and subtypes also stratified by menopausal status. Compared with women diagnosed with ER-negative tumors, women with ER-positive tumors had significantly higher PD and dense area (for PD: 25.8% versus 19.3%, *P* = 0.02; and for dense area: 94.4 versus 63.2 cm^2^, *P* = 0.01) (Table 3). These patterns were consistent albeit attenuated when analyses were stratified by menopausal status. No significant relationships were observed between MBD measurements and either PR or HER2 status. In contrast, TNBC breast cancer cases had significantly lower PD and dense area as compared with non-TNBC subtypes (for PD: 17.4 versus 26.1%, *P* = 0.01, and for dense area: 56.3 versus 96.6 cm^2^, *P* < 0.01), findings that were most apparent, albeit attenuated, among premenopausal women. Overall, no relationships were observed between nondense area and any of the breast cancer molecular subtypes examined.

## 4. Discussion

This study is the first to present data from indigenous Kenyan women showing relationships between breast cancer clinical characteristics and MBD. To date, much of the literature surrounding MBD has focused on Western populations. In this analysis, we show inverse relationships between MBD and patient age, which are consistent with Western trends [7], with higher PD and dense area observed in younger than older women. Similar inverse associations were found for MBD and menopausal status, with higher MBD observed among premenopausal compared with postmenopausal women. In contrast to Western populations of screened women [10, 19, 20], we did not identify relationships between MBD measurements and breast cancer prognostic features such as tumor size, grade, and nodal status. However, we did find significant differences in MBD measures by molecular tumor subtype, with higher PD and absolute dense area for women who were positive for ER expression and lower PD and absolute dense area for women with TNBC.

As highlighted in the Introduction section, a prior analysis of MBD among Ugandan women referred for mammographic screening and without breast cancer found that a large proportion of study participants had low breast density (BI-RADS category 1 and 2; 67.9%) [11]. This proportion is higher than studies among Western populations, including a study of MBD among patients undergoing a breast biopsy following an abnormal mammogram which found that about 40% of African-American patients had MBD of either BI-RADs category 1 or 2, which roughly reflects PD < 50% [14]. Within our study population, the mean PD was 26.6%, with most (89%) women having PD measurements less than 50%. These distributions are comparable to those found among the screening population in Uganda, although BI-RADs measurements were utilized in Uganda. Studies that have examined the relationship between MBD and race have found mixed results to date and often yielded differing findings following the inclusion of age, BMI, and breast size as adjustments factors, all of which vary between populations [13]. The high proportion of lower breast density observed in SSA could be partly accounted for by elevated BMI, as an increase in breast size associated with a higher BMI may result in lower percentage breast density. However, BMI was not reported in the prior Ugandan study and thus a strength of this study was our ability to account for this influence by including BMI as an adjustment factor within our analysis.

Among breast cancer patients within SSA, it is recognized that there are differences in breast cancer characteristics compared with Western populations. Findings of a recent meta-analysis by Jedy-Agba and colleagues, which included 83 studies from 17 countries in SSA and reviewed breast cancer diagnosis stage in this setting, found that 77% of breast cancer patients were diagnosed with stage 3/4 disease and 10–15 years younger than in developed countries [21, 22]. In agreement with that meta-analysis, our study population had a high proportion of tumors that were larger, higher grade, and positive for lymph node metastasis. Our study showed no association between these prognostic characteristics and MBD measurements. While to our knowledge these relationships have not been previously investigated in Kenya, these findings contrast with a larger case-control study nested within the Nurses' Health Study prospective cohort, which found that elevated MBD was associated with larger, higher grade tumors [20].

The literature investigating relationships between MBD and breast cancer molecular subtypes defined according to hormone receptor status have also been inconsistent to date. A meta-analysis conducted by Antoni and colleagues that is consisted of over 24,000 breast cancer cases from 19 studies found that the magnitudes of the relationships between MBD and risk of ER-positive and ER-negative breast cancer were similar, highlighting that MBD is a strong marker of breast cancer risk irrespective of clinical subtype [8]. However, it is important to note that the majority of the studies included in that meta-analysis were based within US and European populations [8]. We showed higher MBD measurements among women with ER-positive than ER-negative breast cancers, findings that were attenuated when stratified by menopausal status. While these findings could be an artifact of a smaller sample size of ER-negative breast cancers, these relationships are likely influenced by multiple factors that could affect both MBD and ER-status, including menopausal status [23]. Of note in this study, a higher proportion of ER-positive compared to ER-negative breast cancer cases was premenopausal at diagnosis. MBD is inversely associated with menopausal status and is higher in younger premenopausal women in this and other populations [23]. In analyses stratified by menopausal status, differences in MBD by ER status were attenuated among premenopausal women. Future studies with larger numbers of ER-positive and ER-negative breast cancers from pre- and postmenopausal women are needed to clarify these relationships among African women. The findings observed for lower PD and absolute dense area among women with TNBC are consistent with our findings of higher MBD measurements observed for women positive for ER expression.

A high proportion of low MBD in SSA, particularly among indigenous Kenyan women may have important implications for the application of cancer prevention initiatives. For example, lower resource settings, where it may not be feasible to establish large population-based screening programs for breast cancer, may obtain greater benefits from inexpensive and relatively safe breast cancer screening tools, such as mammography or ultrasound. This is particularly relevant in a resource challenged setting like Kenya, where breast cancer remains the most common cancer of women, with an age-standardized incidence rate of 51.7 per 100,000 [24].

This analysis is one of the first to date to investigate the role of MBD among indigenous Kenyan women with breast cancer. In addition, the use of computer-assisted software for breast density assessment allows for quantitative interpretation of MBD measures, reducing the potential biases associated with visual assessment. The population included in this analysis was not enrolled in a breast cancer screening program and thus their diagnoses were based upon presentation of symptoms or evaluation of breast lumps. Therefore, this provides a unique opportunity to examine relationships between MBD and breast tumor molecular subtypes without the influence of screening. Limited data, however, were available on traditional breast cancer risk factors including family history and reproductive factors such as parity. Therefore, future studies will be needed to determine whether the observed relationships between MBD and ER-positive versus ER-negative breast cancer are influenced by these factors. Expanded ongoing efforts in Kenya to understand the role of MBD in breast cancer risk and progression will further complement this analysis. Kenya's participation in the International Consortium of Mammographic Density, a consortium developed by the International Agency for Research on Cancer (IARC), whose goal is to pool international MBD data on women without breast cancer, will allow for increased understanding of the role of MBD in breast cancer etiology within this population and will also allow in-depth investigation of MBD in Kenya and its comparability with other countries [25].

It is acknowledged that the landscape of breast cancer in Africa is changing with increased incidence and mortality being observed over the last 20 years. As the drivers of these trends are not fully elucidated, it is important to better understand factors that may influence breast tumor development and progression. This study is a first step in understanding MBD from a Kenyan and African perspective in a clinical setting. The differential patterns of association observed within this population for MBD by ER status suggest that MBD contributes to ER-positive and ER-negative breast cancers potentially through differential mechanisms. Identifying factors specific to breast cancer molecular subtypes will help improve our understanding of ER-positive versus ER-negative tumor etiology and progression.

In conclusion, we present results from indigenous Kenyan breast cancer patients that offer further insights into the role of MBD in breast cancer. This study provides a foundation for expanding efforts to investigate the role of MBD in breast cancer risk and prognosis in Kenya and to understand geographical variation in MBD between Kenya and other countries worldwide. Additional larger multicenter studies in independent validation populations are needed to confirm these findings.

## Figures and Tables

**Table 1:** Select characteristics of breast cancer patients with pretreatment diagnostic mammograms, Aga Khan University Hospital, Kenya, 2014-2015.

Clinical characteristic	Overall (*n* = 123)		ER-positive (*n* = 90)		ER-negative (*n* = 33)	
	*n*	(%)	*n*	(%)	*n*	(%)
Age						
<50 years	48	39	37	41	11	33
≥50 years	75	61	53	59	22	67
Mean (years), SD	54 (11)		53 (11)		57 (12)	
Median (range)	54 (27-83)		54 (27-81)		54 (39-83)	
Menopausal status						
Pre	42	34	33	37	9	27
Post	81	66	57	63	24	73
BMI category						
Lean	18	15	13	14	5	15
Overweight	54	44	40	44	14	42
Obese	43	35	32	36	11	33
Missing	8	7	5	6	3	9
Mean (kg/m^2^), SD	29 (4.8)		29 (4.7)		28 (5.3)	
Median (range)	28 (18-46.8)		28 (19-45.6)		28 (18-46.8)	
Diagnosis						
Invasive ductal	105	85	77	86	28	85
Other	18	15	13	14	5	15
Tumor size						
<3 cm	53	43	44	49	9	27
≥3 cm	58	47	39	43	19	58
Missing	12	10	7	8	5	15
Mean (cm), SD	3.3 (1.8)		3.3 (1.8)		3.4 (1.9)	
Median (range)	3 (0.5-10)		2.7 (1-10)		3 (0.5-8)	
Tumor grade						
Grade 1	14	11	13	14	1	3
Grade 2	57	46	51	57	6	18
Grade 3	49	40	25	28	24	73
Missing	3	2	1	1	2	6
Nodal status						
Negative	49	40	36	40	13	39
Positive	60	49	46	51	14	42
Missing	14	11	8	9	6	18
PR status						
Negative	40	33	9	10	31	94
Positive	83	67	81	90	2	6
HER2 status						
Negative	94	76	71	79	23	70
Positive	22	18	14	16	8	24
Equivocal/DCIS	7	6	5	6	2	6
TNBC status						
Negative	95	77	85	94	10	30
Positive	21	17	0	0	21	64
Missing	7	6	5	6	2	6

BMI: body mass index; ER: estrogen receptor; HER2: human epidermal growth factor receptor 2; PR: progesterone receptor; TNBC: triple negative breast cancer.

**Table 2:** Relationships between patient and clinical characteristics of women with breast cancer and mammographic density measurements (unadjusted and adjusted analysis).

Clinical characteristic	*n*	(%)	Percent density (%)						Absolute dense area						Nondense area					
			Unadjusted			Adjusted^*∗*^			Unadjusted			Adjusted^*∗*^			Unadjusted			Adjusted^*∗*^		
			Mean	95% CI		Mean	95% CI		Mean	95% CI		Mean	95% CI		Mean	95% CI		Mean	95% CI	
Age																				
<50 years	48	39	31.9	28.6	35.2	31.8	28.4	35.2	106.2	91.5	120.9	110.1	95.0	125.3	223.8	189.3	258.4	232.5	200.3	264.8
≥50 years	75	61	19.9	17.8	22.0	19.8	17.7	21.9	70.7	61.1	80.2	72.1	62.4	81.7	301.6	269.5	333.7	306.9	277.7	336.0
***P value***			***<0.01***			***<0.01***			***<0.01***			***<0.01***			***0.03***			***0.02***		
Menopausal status																				
Pre	42	34	30.7	27.1	34.2	24.0	19.9	28.0	96.2	80.9	111.5	69.1	52.8	85.4	214.1	178.2	250.1	228.8	184.8	272.8
Post	81	66	21.2	19.0	23.3	24.1	21.6	26.7	77.5	67.6	87.4	94.7	82.7	106.7	301.4	270.6	332.1	303.1	271.3	334.9
***P value***			***<0.01***			*0.96*			*0.15*			*0.13*			***0.01***			*0.10*		
BMI category																				
Lean	18	15	27.0	21.7	32.2	26.2	21.4	31.0	50.8	34.3	67.3	49.1	33.3	64.8	133.8	95.1	172.5	137.0	98.6	175.5
Overweight	54	44	25.4	22.5	28.4	24.9	22.2	27.6	86.1	73.6	98.6	84.5	72.5	96.5	261.9	230.4	293.3	265.1	234.1	296.2
Obese	43	35	21.3	18.3	24.3	22.2	19.3	25.1	102.9	87.6	118.2	106.1	91.0	121.2	376.0	333.8	418.2	368.8	327.6	409.9
***P value for trend***			*0.29*			*0.52*			***0.01***			***<0.01***			***<0.01***			***<0.01***		
Diagnosis																				
Invasive ductal	105	85	24.6	22.5	26.6	24.1	22.1	26.0	80.7	71.8	89.6	82.8	74.3	91.4	256.7	231.5	281.9	268.0	245.2	290.8
Other	18	15	22.6	17.7	27.4	23.9	18.9	28.9	101.8	77.7	125.9	106.2	81.2	131.2	353.4	282.1	424.7	342.3	275.8	408.8
***P value***			*0.59*			*0.94*			*0.25*			*0.21*			*0.07*			*0.14*		
Tumor size																				
<3 cm	53	43	22.7	19.9	25.6	22.1	19.4	24.7	72.5	60.8	84.1	74.4	63.3	85.5	254.5	218.6	290.4	271.3	238.5	303.1
≥3 cm	58	47	24.8	22.0	27.7	24.9	22.2	27.7	91.5	79.0	104.1	93.2	80.9	105.5	289.9	253.3	326.5	283.6	255.6	321.6
***P value***			*0.47*			*0.30*			*0.13*			*0.12*			*0.34*			*0.60*		
Tumor grade																				
Grade 1	14	11	31.4	25.0	37.9	30.5	24.6	36.4	107.3	78.9	135.7	118.7	91.1	146.4	253.2	184.8	321.5	295.5	230.5	360.4
Grade 2	57	46	22.8	20.1	25.6	22.9	20.3	25.5	82.0	69.7	94.3	88.2	76.1	100.3	280.3	244.5	316.1	294.4	261.5	327.4
Grade 3	49	40	24.8	21.7	27.8	24.1	21.3	27.0	78.8	65.8	91.9	74.2	62.2	86.1	247.5	211.2	283.8	242.0	209.9	274.2
***P value for trend***			*0.21*			*0.25*			*0.43*			*0.10*			*0.65*			*0.26*		
BMI: body mass index																				
*Nodal status*																				
Negative	49	40	24.8	21.6	27.9	24.1	21.1	27.0	92.1	78.2	106.0	91.6	78.3	104.9	297.9	257.8	337.9	298.4	263.1	333.8
Positive	60	49	22.9	20.2	25.6	22.9	20.3	25.4	76.1	64.7	87.5	79.4	68.5	90.3	257.2	223.5	290.8	270.3	240.7	299.8
***P value***			*0.53*			*0.67*			*0.22*			*0.33*			*0.28*			*0.40*		

^*∗*^Adjusted for age and BMI. BMI: body mass index.

**Table 3:** Relationship between MD and tumor subtype (overall and stratified by menopausal status) among women included in this study.

Clinical characteristic	*n*	(%)	Percent density (%)						Absolute dense area						Nondense area					
			Unadjusted			Adjusted^*∗∗*^			Unadjusted			Adjusted^*∗∗*^			Unadjusted			Adjusted^*∗∗*^		
			Mean	95% CI		Mean	95% CI		Mean	95% CI		Mean	95% CI		Mean	95% CI		Mean	95% CI	
*All women*																				
ER status																				
Negative	33	27	20.0	16.7	23.3	19.3	16.2	22.4	61.2	47.7	74.7	63.2	49.9	76.6	280.3	232.7	327.8	286.0	242.7	329.3
Positive	90	73	25.9	23.7	28.2	25.8	23.7	28.0	92.8	82.7	102.9	94.4	84.8	104.1	266.1	238.0	294.2	274.1	249.0	299.2
*P value *			***0.04***			***0.02***			***0.01***			***0.01***			*0.73*			*0.76*		
*Premenopausal women*																				
ER status																				
Negative	9	21	24.5	17.5	31.6	25.0	16.7	33.4	73.6	44.1	103.1	63.3	31.0	95.6	249.2	178.4	320.0	216.4	140.7	292.1
Positive	33	79	32.5	28.3	36.8	32.2	27.7	36.7	103.4	85.0	121.8	104.8	85.0	124.6	205.4	171.7	239.1	209.7	174.5	244.8
*P value *			*0.19*			*0.31*			*0.25*			*0.16*			*0.45*			*0.95*		
*Postmenopausal women*																				
ER status																				
Negative	24	30	18.4	14.9	22.0	17.0	13.8	20.1	57.3	42.4	72.3	64.4	50.5	78.4	293.6	233.6	353.5	336.8	283.5	390.2
Positive	57	70	22.5	20.0	25.0	22.9	20.6	25.3	87.2	75.1	99.2	88.6	78.0	99.3	305.4	265.7	345.2	304.5	271.5	337.5
*P value *			*0.20*			*0.05*			***0.04***			*0.07*			*0.81*			*0.48*		
*All women*																				
PR status																				
Negative	40	33	22.0	18.9	25.2	22.4	19.2	25.5	65.0	52.3	77.7	71.4	58.2	84.5	250.9	210.1	291.7	259.2	221.6	296.9
Positive	83	67	25.4	23.0	27.7	24.8	22.6	27.1	93.5	82.9	104.1	92.7	82.6	102.7	279.3	249.4	309.2	285.6	259.0	312.2
*P value *			*0.25*			*0.38*			***0.02***			*0.08*			*0.43*			*0.43*		
*Premenopausal women*																				
PR status																				
Negative	11	26	26.0	19.4	32.6	28.1	19.9	36.3	78.4	50.7	106.1	78.1	44.7	111.5	241.4	178.2	304.6	217.0	148.1	285.9
Positive	31	74	32.5	28.1	36.9	31.7	27.0	36.4	103.5	84.4	122.5	102.5	81.6	123.4	205.3	170.5	240.1	209.1	172.4	245.8
*P value *			*0.27*			*0.59*			*0.31*			*0.40*			*0.50*			*0.92*		
*Postmenopausal women*																				
PR status																				
Negative	29	36	20.7	17.3	24.1	19.5	16.3	22.7	60.6	46.6	74.7	68.5	55.2	81.9	255.4	205.3	305.6	296.9	250.8	343.0
Positive	52	64	21.6	18.9	24.2	21.9	19.5	24.4	88.1	75.4	100.8	88.2	77.1	99.3	329.5	286.9	372.1	323.4	288.1	358.7
*P value *			*0.77*			*0.41*			*0.05*			*0.12*			*0.13*			*0.53*		
*All women*																				
TNBC status																				
No	95	77	25.8	23.5	28.0	26.1	23.9	28.2	92.9	83.1	102.7	96.6	87.4	105.7	271.3	244.6	298.1	279.2	255.5	302.8
Yes	21	17	20.1	15.9	24.3	17.4	13.6	21.1	58.7	42.2	75.2	56.3	41.1	71.4	266.5	210.2	322.8	287.2	234.9	339.4
*P value *			*0.11*			***0.01***			***0.02***			***<0.01***			*0.89*			*0.87*		
*Premenopausal women*																				
TNBC status																				
No	33	85	33.1	28.7	37.4	33.1	28.6	37.6	111.6	93.9	129.3	115.7	97.9	133.5	218.9	187.8	250.0	225.0	193.6	256.4
Yes	6	15	22.0	13.7	30.2	19.0	10.4	27.5	54.1	25.7	82.5	37.3	12.5	62.1	222.9	149.7	296.1	216.7	139.3	294.1
*P value *			*0.12*			*0.07*			***0.04***			***0.01***			*0.98*			*0.85*		
*Postmenopausal women*																				
TNBC status																				
No	62	81	22.3	19.8	24.7	22.9	20.6	25.2	83.8	72.2	95.4	87.6	77.2	98.0	301.9	264.2	339.7	306.7	274.4	339.1
Yes	15	19	19.5	14.9	24.1	16.4	12.4	20.4	61.4	41.4	81.4	64.0	45.8	82.2	286.7	212.1	361.3	331.9	262.9	400.9
*P value *			*0.46*			*0.07*			*0.19*			*0.13*			*0.78*			*0.66*		
*All women*																				
HER2 status																				
Negative	94	76	24.2	22.0	26.4	23.8	21.7	25.8	83.1	73.6	92.6	86.4	77.4	95.5	270.2	243.3	297.1	284.8	260.8	308.8
Positive	22	18	26.9	22.1	31.7	27.4	22.8	32.1	99.5	78.1	121.0	99.3	78.9	119.7	271.4	215.9	327.0	262.0	213.6	310.5
***P value***			*0.49*			*0.32*			*0.33*			*0.43*			*0.99*			*0.55*		

^*∗∗*^Adjusted for age and BMI and additionally adjusted for menopausal status in analyses that included all women. BMI: body mass index; ER: estrogen receptor; HER2: human epidermal growth factor receptor 2; PR: progesterone receptor; TNBC: triple negative breast cancer; CI: confidence intervals.

## Supplementary Materials

Supplementary Materials Supplementary Figure  1. Distribution of mammographic breast density (MBD) measurements by age including (A) dense area, (B) nondense area, and (C) breast area.
