# Supplementary material for: Mammographic Breast Density and Breast Cancer Molecular Subtypes: The Kenyan-African Aspect
Source: Biomed Res Int. 2018 Jan 22;2018:6026315. doi: 10.1155/2018/6026315 (PMC5828539; doi:10.1155/2018/6026315)

Supplementary Figure 1: Distribution of mammographic breast density (MBD) measurements by age including (A) dense area, (B) non-dense area and (C) breast area

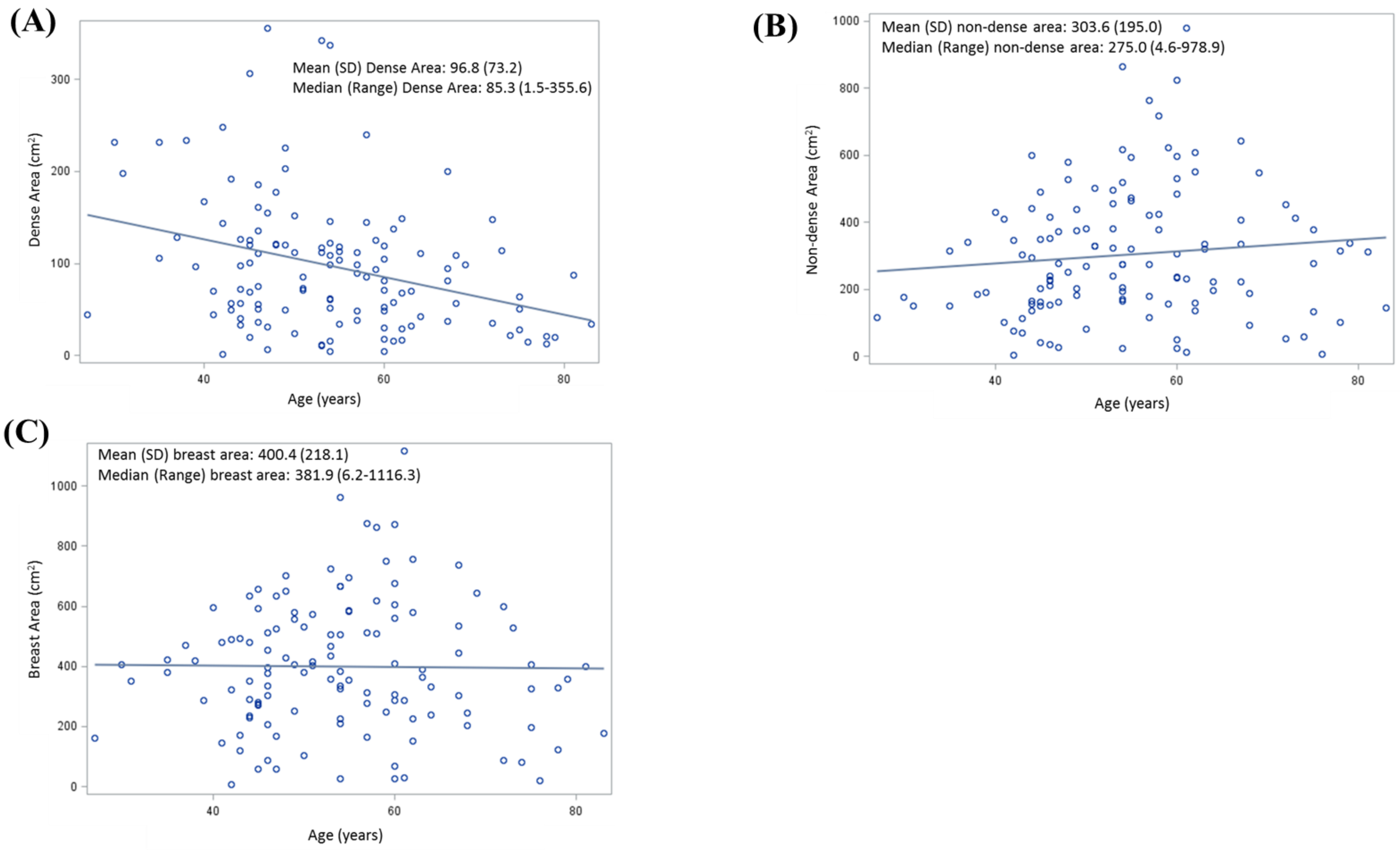

Supplement: Supplementary Materials — Supplementary Figure 1. Distribution of mammographic breast density (MBD) measurements by age including (A) dense area, (B) nondense area, and (C) breast area. [file 6026315.f1.pdf]
